# Distribution and prevalence of vector-borne diseases in California chipmunks (*Tamias* spp.)

**DOI:** 10.1371/journal.pone.0189352

**Published:** 2017-12-12

**Authors:** Mary H. Straub, Austin N. Roy, Amanda Martin, Kathleen E. Sholty, Nicole Stephenson, Janet E. Foley

**Affiliations:** 1 Department of Veterinary Medicine and Epidemiology, School of Veterinary Medicine, University of California, Davis, California, United States of America; 2 Museum of Wildlife and Fish Biology, University of California, Davis, California, United States of America; 3 Wildlife Investigations Laboratory, California Department of Fish and Wildlife, Rancho Cordova, California, United States of America; University of Maryland, College Park, UNITED STATES

## Abstract

California, with 13 chipmunk (*Tamias*) species, has more than any other state or country, occupying habitats ranging from chaparral to the high peaks of the Sierra Nevada. Chipmunks host zoonotic pathogens including *Yersinia pestis*, *Anaplasma phagocytophilum*, relapsing fever (RF) *Borrelia* spp., *Borrelia burgdorferi*, and spotted fever group (SFG) *Rickettsia* species. Chipmunk species are often not differentiated by public health workers, yet different species utilize different ecological niches and may have intrinsically different capacities for maintaining vector-borne pathogens and infecting vectors. We surveyed over 700 individuals from nine species of chipmunks throughout California for exposure to and infection by *Y*. *pestis*, *A*. *phagocytophilum*, RF *Borrelia* spp., *Borrelia burgdorferi*, and SFG *Rickettsia* species. DNA of all five pathogens was found and all chipmunks except Merriam’s chipmunk (*T*. *merriami*) were PCR-positive for at least one of the pathogens. *Anaplasma phagocytophilum* was most common (40.0%, 2/5) in Sonoma chipmunks (*T*. *sonomae*) from Marin county and *B*. *burgdorferi* most common (37.5%, 27/72) in redwood chipmunks (*T*. *ochrogenys*) from Mendocino county. RF *Borrelia* spp. was detected in 2% (6/297) of redwood chipmunks in Mendocino county and 10% (1/10) of both least (*T*. *minimus*) and lodgepole (*T*. *speciosus*) chipmunks in the western Sierra. Exposure to SFG *Rickettsia* spp. was found in the Northern Coastal region (Del Norte, Humboldt and Mendocino counties) and in the northern and western Sierra in several species of chipmunks. *Y*. *pestis* infection was found only in the western Sierra—in a yellow-pine (*T*. *amoenus*) and a long-eared (*T*. *quadrimaculatus*) chipmunk. Though more data are needed to thoroughly understand the roles that different chipmunk species play in disease transmission, our findings suggest that some chipmunk species may be more important to the maintenance of vector-borne diseases than others within each geographic area.

## Introduction

Chipmunks (*Tamias* spp.) are small, terrestrial, diurnal mammals characterized as a genus by rapid reproductive rates and high population densities [1]. There are 25 species in the world, 24 in North America, and 23 in western North America, including two found only in Mexico. Though the status of most chipmunk species is stable, Palmer’s chipmunk (*T*. *palmeri*) is federally endangered [2] and Buller’s chipmunk (*T*. *bulleri*) is classified as vulnerable by the IUCN [3].

Chipmunks host important zoonotic pathogens in the western United States including *Yersinia pestis*, the causative agent of plague, *Anaplasma phagocytophilum*, which causes granulocytic anaplasmosis, relapsing fever (RF) *Borrelia* spp., *Borrelia burgdorferi*, the agent of Lyme disease, and spotted fever group (SFG) *Rickettsia* species [4–8]. *Y*. *pestis* is potentially fatal in chipmunks as well as humans [9], while *A*. *phagocytophilum*, RF *Borrelia* spp., *B*. *burgdorferi* and SFG *Rickettsia* spp. are zoonoses but not known to cause clinical disease in chipmunks [10–13].

In California, which is home to 13 species of chipmunks, chipmunks occupy diverse habitats ranging from coastal chaparral which is home to Merriam’s chipmunk (*T*. *merriami*), to the high peaks of the Sierra Nevada where the alpine chipmunk (*T*. *alpinus*) can be found. In the eastern-central Sierra Nevada, ranges of the lodgepole (*T*. *speciosus*), least (*T*. *minimus*), yellow-pine (*T*. *amoenus*), Allen’s (*T*. *senex*), and Uinta chipmunks (*T*. *umbrinus*) overlap at elevations between approximately 1800 and 2700m. On the western slope of the central Sierra Nevada, the long-eared (*T*. *quadrimaculatus*), lodgepole, Allen’s and Merriam’s chipmunk are found at elevations close to 1800m [14, 15]. (For distribution maps for all California chipmunk species, see pages 210–225 of [15] and for maps depicting the overlaps of Sierra Nevada chipmunk species, see pages 315–317 of [14].) Chipmunks also share vector-borne pathogens with woodrats (*Neotoma* spp.), deer mice (*Peromyscus* spp.), and other sciurids such as the western grey squirrel (*Sciurus griseus*), California ground squirrel (*Otospermophilus beecheyi*), and Douglas squirrel (*Tamiasciurus douglasii*) [4, 16–23].

Chipmunk species can be difficult for the lay-person to differentiate and are often referred to generically in the public health arena, without differentiating by species [8, 9, 24]. However, different species utilize different ecological niches, which may lead to varying exposure to vectors and vector-borne pathogens. Additionally, host competence for vector-borne pathogens may vary among chipmunk species. The current understanding of vector-borne diseases in chipmunks in the western US does not adequately fill these gaps in knowledge.

In order to address these gaps, we undertook a survey in chipmunks for the vector-borne pathogens *A*. *phagocytophilum*, *B*. *burgdorferi*, RF *Borrelia* spp. SFG *Rickettsia* spp. and *Y*. *pestis*, sampling over 700 individuals from nine species of chipmunks throughout California. We evaluated for differences in infection and exposure prevalence by geographic region and by chipmunk species, and we investigated relationships between prevalence and chipmunk species diversity and richness at each site. Test agreement between evidence of exposure and active infection was also explored.

## Materials and methods

### Ethics statement

Trapping and sampling of chipmunks (*Tamias* spp.) were covered under California Department of Fish and Wildlife scientific collecting permit #SC-854. The study spanned multiple applicable protocols which were approved by the University of California, Davis Institutional Animal Care and Use Committee. The most recent protocol is #18179.

### Trapping and sample collection

Chipmunks were live-trapped using Sherman (HB Sherman, Tallahassee, FL, USA) or Tomahawk (Tomahawk Live Trap, Hazlehurst, WI, USA) traps baited with oats and peanut butter or molasses and birdseed. Trapping took place opportunistically at 16 study sites distributed across 12 California counties (Alpine, El Dorado, Humboldt, Madera, Marin, Mendocino, Mono, Nevada, Placer, Santa Cruz, Sierra, Tuolumne) between October 2005 and August 2015. Animals were trapped both during daytime and nighttime hours and traps were checked early morning, twice during the day and in the late evening. Animals were anesthetized with up to 40mg/kg ketamine and 4mg/kg xylazine delivered subcutaneously. Each animal was identified to species in the field when possible, and the age (juvenile or adult) and sex were determined. Blood was collected from the retroorbital sinus or the lateral saphenous vein into EDTA blood collection tubes (Becton, Dickinson and Company, Franklin Lakes, NJ, USA) and kept cool until plasma was separated by centrifugation at 3000rpm for 10 minutes. Whole blood and plasma were then stored at -20°C to -80°C until use. A small piece of marginal ear tissue (2mm^2^) was taken using sterile scissors, stored in 70% ethanol, and kept cool until it could be refrigerated. (Ear tissue samples were collected routinely starting in 2008 so were not available for all chipmunks.) Each animal was given an individually numbered metal ear tag prior to release. Additional museum samples were collected by US Forest Service in 2010, 2012 and 2013 in the western Sierra Nevada (El Dorado, Madera, Tuolumne and southern Placer counties) and donated to the UC Davis Museum of Wildlife and Fish Biology. Blood and ear snips were collected from these chipmunks post-mortem. All work with animals was performed in compliance with the UC Davis or Humboldt State University IACUC, and under valid scientific collecting permits from California Department of Fish and Wildlife, the U.S. Forest Service, and landowners where applicable.

### Genetic determination of species

When species could not be differentiated in the field, amplification of the *cytochrome b* gene was performed by PCR using primers MVZ05 and MVZ16 [25] and thermocycling conditions as previously described [26]. PCR products were electrophoresed on 1% agarose gels stained with Gelstar (Lonza Inc., Allendale, NJ, USA). Amplified DNA bands were visualized using UV transillumination, excised with x-tracta Gel Extractors (Promega, Madison, WI, USA), purified using a kit (Qiaquick Gel Extraction Kit, Qiagen, Valencia, CA, USA), and sequenced in an ABI Prism 3730 sequencer (UC DNA Sequencing Facility, Davis, CA, USA). Results were examined for accuracy of base determination and end-read errors were trimmed to yield unambiguous sequences. Sequences were compared to those in the Genbank database using the Basic Local Alignment Search Tool (BLAST, National Center for Biotechnology Information, Bethesda, MD, USA).

### Serology

Indirect immunofluorescent assays (IFA) were performed to evaluate for exposure to *A*. *phagocytophilum*, *Borrelia* spp. and SFG *Rickettsia* spp. Plasma was diluted 1:25 in phosphate-buffered saline (PBS) and then applied to *A*. *phagocytophilum*, *B*. *burgdorferi* or *R*. *rickettsii* antigen slides (VMRD, Pullman, WA, USA). Slides were incubated at 37°C with moisture for 45 min, washed three times in PBS and then incubated at 37°C for 45 minutes with fluorescein isothiocyanate-labeled rabbit anti-rat immunoglobulin G heavy and light chain antibodies (Kirkegaard & Perry Laboratories, Gaithersburg, MD, USA) diluted in PBS at 1:25. Slides were washed three additional times in PBS and, during the third wash, incubated with two drops of eriochrome black for 2 minutes. Positive (previously tested rodents) and negative (PBS and negative rodent serum) controls were included in each run. Samples were considered positive if fluorescence was detected in the appropriate distribution for each pathogen.

### DNA extraction and PCR

Chipmunks were assessed for active infection by real-time TaqMan PCR. DNA was extracted from 100μl of whole blood (for *A*. *phagocytophilum*, *Y*. *pestis*, RF *Borrelia* spp. and SFG *Rickettsia* spp.) or ear tissue (for *B*. *burgdorferi*) using a kit (DNAeasy Blood and Tissue Kit, Qiagen, Valencia, CA, USA) following manufacturer’s instructions. Real-time PCR for *A*. *phagocytophilum* [27], *Y*. *pestis* [28], RF *Borrelia* spp. [29], *B*. *burgdorferi* [29] and SFG *Rickettsia* spp. [30] was performed as previously described. PCR for *A*. *phagocytophilum*, RF *Borrelia* spp., and SFG *Rickettsia* spp. was not performed on all chipmunks due to insufficient sample volumes. PCR for *B*. *burgdorferi* was performed on all chipmunks from which ear tissue samples were collected. A subset of chipmunks from the north coastal region of California (Mendocino, Humboldt and Del Norte counties) and from the Sierra Nevada was evaluated for active infection by *Y*. *pestis*. These two regions were selected for *Y*. *pestis* surveillance as multiple species of animals, including small mammals, from these areas have been found to be seropositive for this pathogen [31, 32].

### Statistical analyses

Data were maintained in Excel (Microsoft, Redmond, Washington) and analyzed in R (version 3.3.2, R-Development Core Team, http://www.r-project.org). For calculating prevalence, geologically, climatically, and botanically related sites were grouped to include counties north of Lake Tahoe (Nevada and Sierra counties) classified as “northern Sierra”, counties south of Lake Tahoe and west of the Sierra crest classified as “western Sierra” and counties south of Lake Tahoe and east of the Sierra crest classified as “eastern Sierra”. Additionally, to compare pathogen prevalence between the Sierra Nevada mountains and the northern coastal region of California, two super-regions were created for statistical analyses: all Sierra regions were combined into “Sierra Nevada” and all counties north of San Francisco were combined into “north-coastal”. Pearson’s chi-square tests were used to assess prevalence differences between regions and between chipmunk species. Because one species (Allen’s chipmunk) has a range that spans two regions, Fisher’s exact tests were used to assess differences in sero- and PCR-prevalence between Allen’s chipmunks from north-coastal California (Humboldt county) and the Sierra Nevada. Fisher’s exact tests were also used to assess the significance of disease prevalence levels between sympatric chipmunk species with different uses of space (i.e. arboreal, terrestrial, mixed) in the Sierra Nevada. Cohen’s Kappa coefficients were calculated to assess agreement between PCR and serology [33, 34]. Correlations between sero- and PCR-prevalence and chipmunk species richness and chipmunk species diversity (H) were investigated by calculating Pearson product-moment correlations. In addition to simple species richness (S), Menhinick’s index (D) was used as a measure of species richness to incorporate the varying sample sizes obtained from each study site [35, 36] and the Shannon-Wiener Index was used as a measure of chipmunk species diversity. A p-value of < 0.05 was considered significant for all tests.

## Results

A total of 709 chipmunks from nine species was sampled between September 2005 and August 2015 (Table 1). The pathogens for which the most evidence was found were *Borrelia* spp., with 11.5% (41/355) of chipmunks being PCR-positive for *B*. *burgdorferi*, 1.4% (8/591) being PCR-positive for RF *Borrelia*, and 66/308 (21.4%) being seropositive, although the serology cross-reacts among the *Borrelia* species. For other pathogens, 8.4% (50/595) of chipmunks were *A*. *phagocytophilum* PCR-positive and 118/526 (22.4%) were seropositive; 0.7% (4/581) were SFG *Rickettsia* spp. PCR-positive and 110/415 (26.5%) were seropositive; and 0.9% (2/230) were *Y*. *pestis* PCR-positive (serology was not performed for *Y*. *pestis*).

**Table 1:** Number of individual chipmunks (*Tamias* spp.) of each species sampled for vector-borne zoonoses in study regions in California between 2005 and 2015.

Region	Del Norte	Humboldt	Mendocino	Marin	Santa Cruz	Northern Sierra	Western Sierra	Eastern Sierra	Total
Years sampled	2011–2013	2005–2007, 2009, 2011–2014	2005–2015	2005–2007	2005–2008	2005–2006, 2010	2006, 2009, 2010–2013, 2015	2010, 2012	
**Yellow-pine**	-	-	-	-	-	57	31	23	111
**Merriam’s**	-	-	-	-	10	-	-	-	10
**Least**	-	-	-	-	-	7	5	-	12
**Yellow-cheeked**	-	-	311	-	-	-	-	-	311
**Long-eared**	-	-	-	-	-	1	70	-	71
**Allen’s**	-	99	-	-	-	33	7	-	139
**Siskiyou**	21	-	-	-	-	-	-	-	21
**Sonoma**	-	-	-	6	-	-	-	-	6
**Lodgepole**	-	-	-	-	-	5	8	3	16
**Unidentified species**	-	-	-	-	-	-	11	1	12
**Total**	21	99	311	6	10	103	132	27	709

Eastern Sierra = counties south of Lake Tahoe and east of the Sierra crest, Northern Sierra = counties north of Lake Tahoe, Western Sierra = counties south of Lake Tahoe and west of the Sierra crest

Agreement between serological and PCR results was low, with Kappa equal to -0.015 (95% CI: -0.032–0.002) for SFG *Rickettsia* spp., 0.088 (0–0.175) for *A*. *phagocytophilum*, 0.098 (0.001–0.195) for RF *Borrelia* spp., and 0.337 (0.148–0.526) for *B*. *burgdorferi*.

### Regional analyses

**Fig 1:**
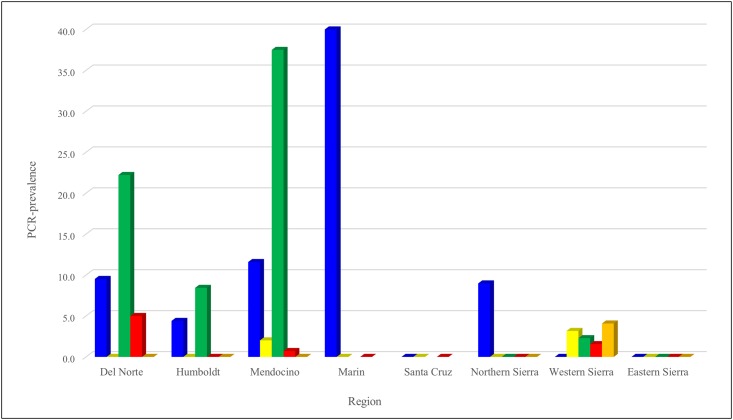
PCR-prevalence of vector-borne zoonotic pathogens in chipmunks (*Tamias* spp.) sampled from eight regions of California between 2005 and 2015. *Anaplasma phagocytophilum* represented by blue. Relapsing fever *Borrelia* spp. represented by yellow. *Borrelia burgdorferi* represented by green. Spotted fever group *Rickettsia* represented by red. *Yersinia pestis* represented by orange.

**Fig 2:**
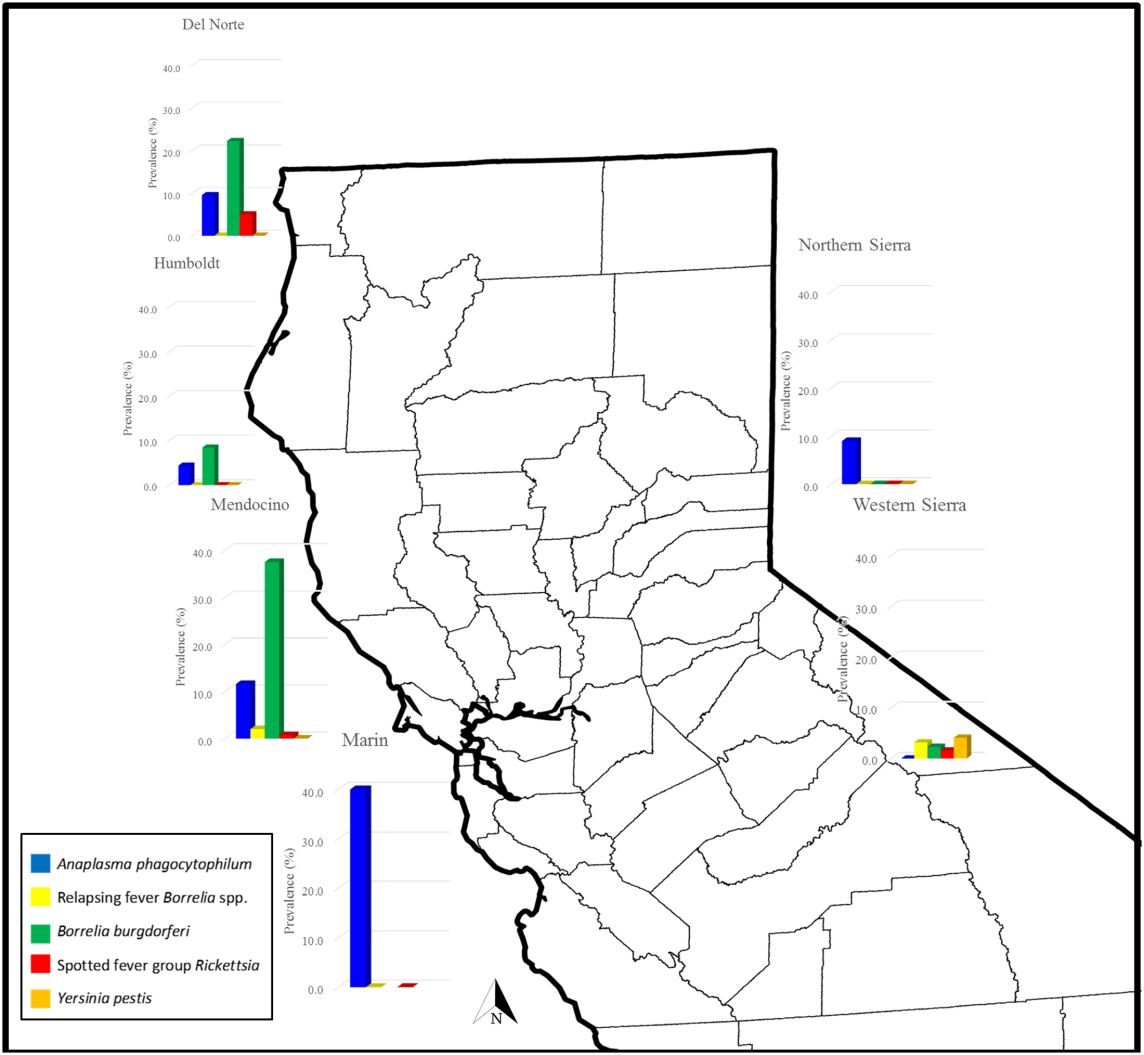
Regional PCR-prevalence of vector-borne zoonotic pathogens in Northern California chipmunks (*Tamias* spp.) sampled between 2005 and 2015. Active infection was not detected in Santa Cruz or the eastern Sierra.

**Fig 3:**
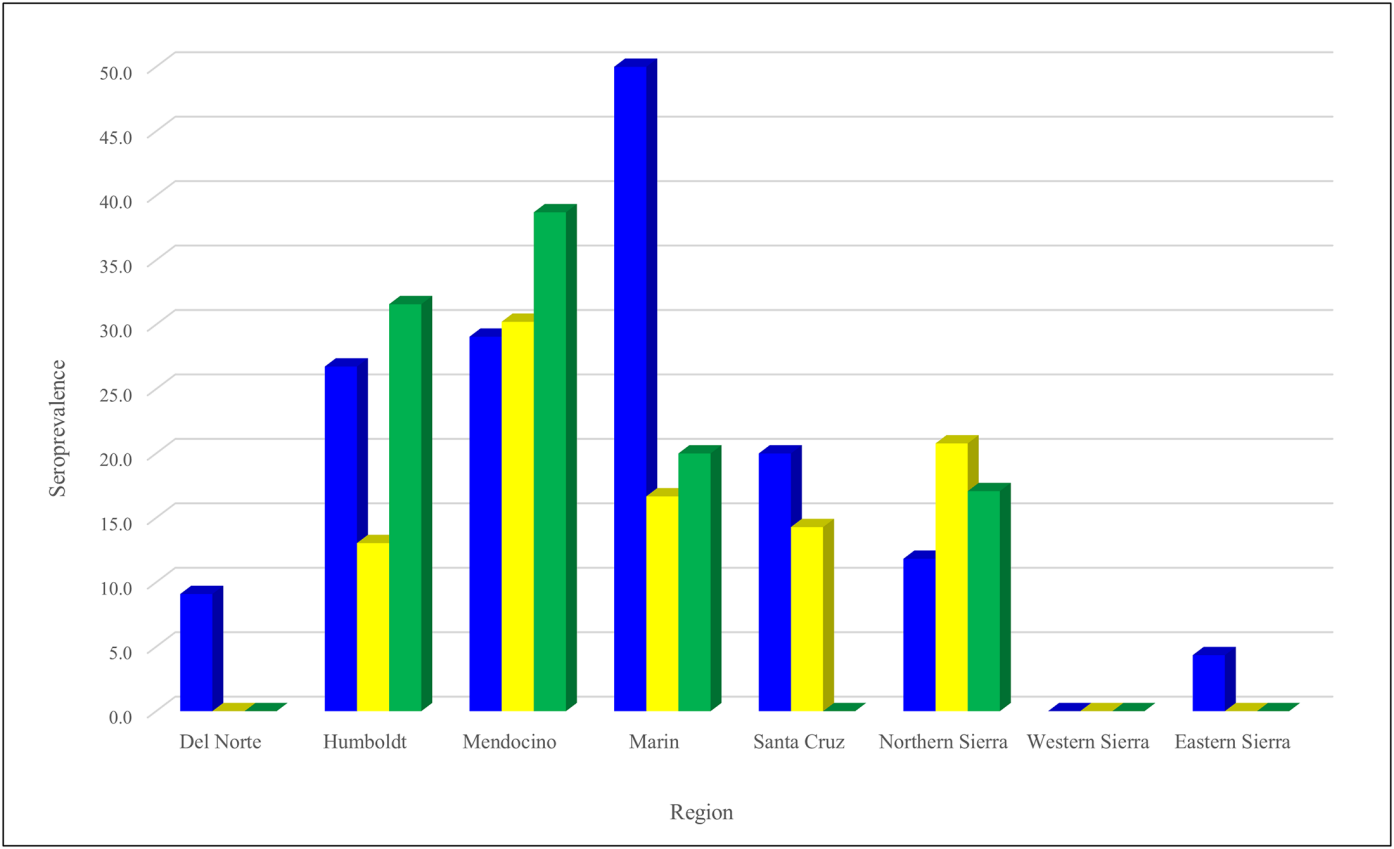
Seroprevalence of vector-borne zoonotic pathogens in chipmunks (*Tamias* spp.) sampled from eight regions of California between 2005 and 2015. *Anaplasma phagocytophilum* represented by blue. *Borrelia* spp. represented by yellow. Spotted fever group *Rickettsia* represented by green.

**Fig 4:**
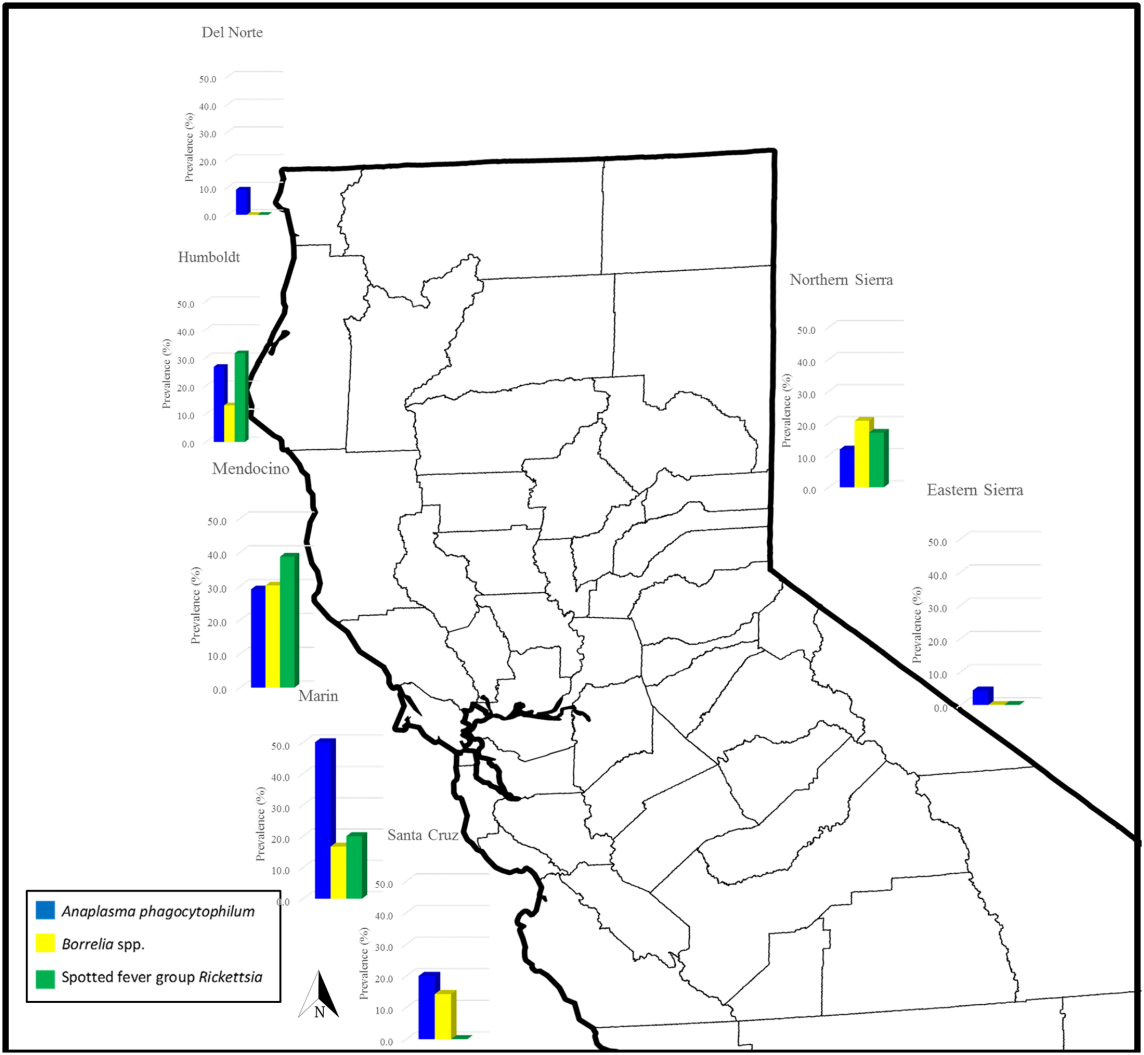
Regional seroprevalence of vector-borne zoonotic pathogens in Northern California chipmunks (*Tamias* spp.) sampled between 2005 and 2015. No evidence of exposure was found in the western Sierra.

In general, regional and species results mirrored each other. Active infection by at least one pathogen was detected in all regions except for Santa Cruz and the eastern Sierra (Table 2, Figs 1 and 2) although there was serological evidence of tick-borne infection in these two regions (Table 3, Figs 3 and 4). Regions differed in *A*. *phagocytophilum* PCR-detection (p = 0.015), with the highest PCR-prevalence in Marin (40.0%, 2/5) and no *A*. *phagocytophilum* PCR-positive results in Santa Cruz or the eastern or western Sierra. On serology, regions again differed significantly (p = 0.004), and the highest seroprevalence was in Marin (50.0%, 3/6), although serological evidence of *A*. *phagocytophilum* was detected in Santa Cruz and the eastern Sierra (Table 3, Figs 3 and 4).

**Table 2:** PCR-prevalence of vector-borne pathogens found in chipmunks (*Tamias* spp.) sampled from eight regions of California between 2005 and 2015.

	Anaplasma phagocytophilum*	Relapsing Fever *Borrelia*	Borrelia burgdorferi*	Spotted Fever Group *Rickettsia*	*Yersinia pestis*
	number positive/number tested (%; 95% CI)	number positive/number tested (%; 95% CI)	number positive/number tested (%; 95% CI)	number positive/number tested (%; 95% CI)	number positive/number tested (%; 95% CI)
**Del Norte**	2/21 (9.5; 2.6–28.9)	0/20 (0.0; 0–16.1)	4/18 (22.2; 15.6–38.6)	1/20 (5.0; 4.0–20.5)	0/19 (0.0; 0–16.9)
**Humboldt**	4/91 (4.4; 1.7–10.8)	0/89 (0.0; 0–4.1)	7/83 (8.4; 7.5–13.0)	0/89 (0.0; 0–4.1)	0/65 (0.0; 0–5.6)
**Mendocino**	35/302 (11.6; 8.5–15.7)	6/297 (2.0; 2.0–3.3)	27/72 (37.5; 31.2–45.1)	2/278 (0.7; 0.7–2.1)	0/60 (0.0; 0–6.0)
**Marin**	2/5 (40.0; 11.8–76.9)	0/5 (0.0; 0–43.4)	n/a	0/5 (0.0; 0–43.4)	n/a
**Santa Cruz**	0/9 (0.0; 0–29.9)	0/5 (0.0; 0–43.4)	n/a	0/10 (0.0; 0–27.8)	n/a
**Northern Sierra**	7/78 (9.0; 4.4–17.4)	0/82 (0.0; 0–4.5)	0/27 (0.0; 0–12.5)	0/89 (0.0; 0–4.1)	0/26 (0.0; 0–12.9)
**Western Sierra**	0/63 (0.0; 0–5.7)	2/63 (3.2; 2.9–8.8)	3/131 (2.3; 2.2–5.1)	1/64 (1.6; 1.5–7.2)	2/49 (4.1; 3.6–11.2)
**Eastern Sierra**	0/26 (0.0; 0–12.9)	0/26 (0.0; 0–12.9)	0/24 (0.0; 0–13.8)	0/26 (0.0; 0–12.9)	0/11 (0.0; 0–25.9)

Eastern Sierra = counties south of Lake Tahoe and east of the Sierra crest, Northern Sierra = counties north of Lake Tahoe, Western Sierra = counties south of Lake Tahoe and west of the Sierra crest. n/a indicates no samples were available from that region for testing. CI = confidence interval.

*A significant difference between regions was found

**Table 3:** Serological results of vector-borne pathogen surveillance of chipmunks (*Tamias* spp.) from eight regions of California sampled between 2005 and 2015.

	Anaplasma phagocytophilum*	*Borrelia* spp.	Spotted Fever Group *Rickettsia*
	number positive/number tested (%; 95% CI)	number positive/number tested (%; 95% CI)	number positive/number tested (%; 95% CI)
**Del Norte**	1/11 (9.1; 1.6–37.7)	0/3 (0.0; 0.0–56.2)	0/8 (0.0; 0.0–32.4)
**Humboldt**	23/86 (26.7; 18.5–37.0)	6/46 (13.0; 10.9–20.9)	24/76 (31.6; 26.4–38.6)
**Mendocino**	77/265 (29.1; 23.9–34.8)	42/139 (30.2; 26.4–35.0)	72/186 (38.7; 34.6–43.4)
**Marin**	3/6 (50.0; 18.8–81.2)	1/6 (16.7; 8.8–50.6)	1/5 (20.0; 9.6–56.5)
**Santa Cruz**	2/10 (20.0; 5.7–51.0)	1/7 (14.3; 8.1–45.8)	0/5 (0.0; 0.0–43.4)
**Northern Sierra**	11/93 (11.8; 6.7–20.0)	16/77 (20.8; 17.6–26.8)	14/82 (17.1; 14.6–22.4)
**Western Sierra**	0/31 (0.0; 0.0–11.0)	0/27 (0.0; 0.0–12.5)	0/28 (0.0; 0.0–12.1)
**Eastern Sierra**	1/23 (4.2; 0.8–21.0)	0/3 (0.0; 0.0–56.2)	0/26 (0.0; 0.0–12.9)

CI = confidence interval. Eastern Sierra = counties south of Lake Tahoe and east of the Sierra crest, Northern Sierra = counties north of Lake Tahoe, Western Sierra = counties south of Lake Tahoe and west of the Sierra crest

*A significant difference was found between regions.

Infection with *B*. *burgdorferi* was found in the north-coastal regions tested (Del Norte, Humboldt and Mendocino; Marin did not have available samples) and in the western Sierra, with a significantly higher PCR-prevalence (37.5%, 27/72) detected in Mendocino (p < 0.0001, Table 2). Relapsing fever *Borrelia* spp. DNA was found only in chipmunks in Mendocino and the western Sierra with no significant difference among regions (p = 0.590). There was evidence of exposure to *Borrelia* spp. in all northwestern regions and the northern Sierra; Mendocino had the highest seroprevalence (30.2%, 42/139), although the difference between regions was not significant (p = 0.086).

Spotted fever group *Rickettsia* spp. were found only in a few individuals sampled in Del Norte, Mendocino and the western Sierra, with Del Norte having the highest PCR-prevalence (5.0%, 1/20), and no significant differences among regions (p = 0.393). Mendocino had the highest seroprevalence (38.7%, 72/186), with additional seropositive animals in Humboldt, Marin, Mendocino, and the northern Sierra; differences in seroprevalence were significant (p = 0.0002). Two individuals from the western Sierra were PCR-positive for *Y*. *pestis*.

When analyzed as a whole, chipmunks from the north-coastal super-region (Mendocino, Humboldt, Del Norte, plus Marin for *A*. *phagocytophilum*) had significantly higher levels of infection with both *A*. *phagocytophilum* and *B*. *burgdorferi* when compared to chipmunks sampled from the Sierra Nevada (p = 0.026 for *A*. *phagocytophilum* and p < 0.0001 for *B*. *burgdorferi*) but no significant differences for PCR-prevalence of RF *Borrelia*, SFG *Rickettsia* spp. or *Y*. *pestis*. Seropositivity for *A*. *phagocytophilum*, *Borrelia* spp. and SFG *Rickettsia* spp. was also higher in the north-coastal super-region than the Sierra Nevada (p < 0.0001).

Chipmunk species diversity ranged from a low of zero in regions where only one chipmunk species was found to a high of 1.14 in the western Sierra. Simple species richness (S) ranged from one to five with the highest number of chipmunk species being found in the northern and western Sierra, while Menhinick’s index (D) ranged from a low of 0.06 in Mendocino to a high of 0.49 in the Northern Sierra. No significant correlations were found between disease prevalence and either chipmunk species richness, S or D, or chipmunk species diversity.

### Species analysis

**Fig 5:**
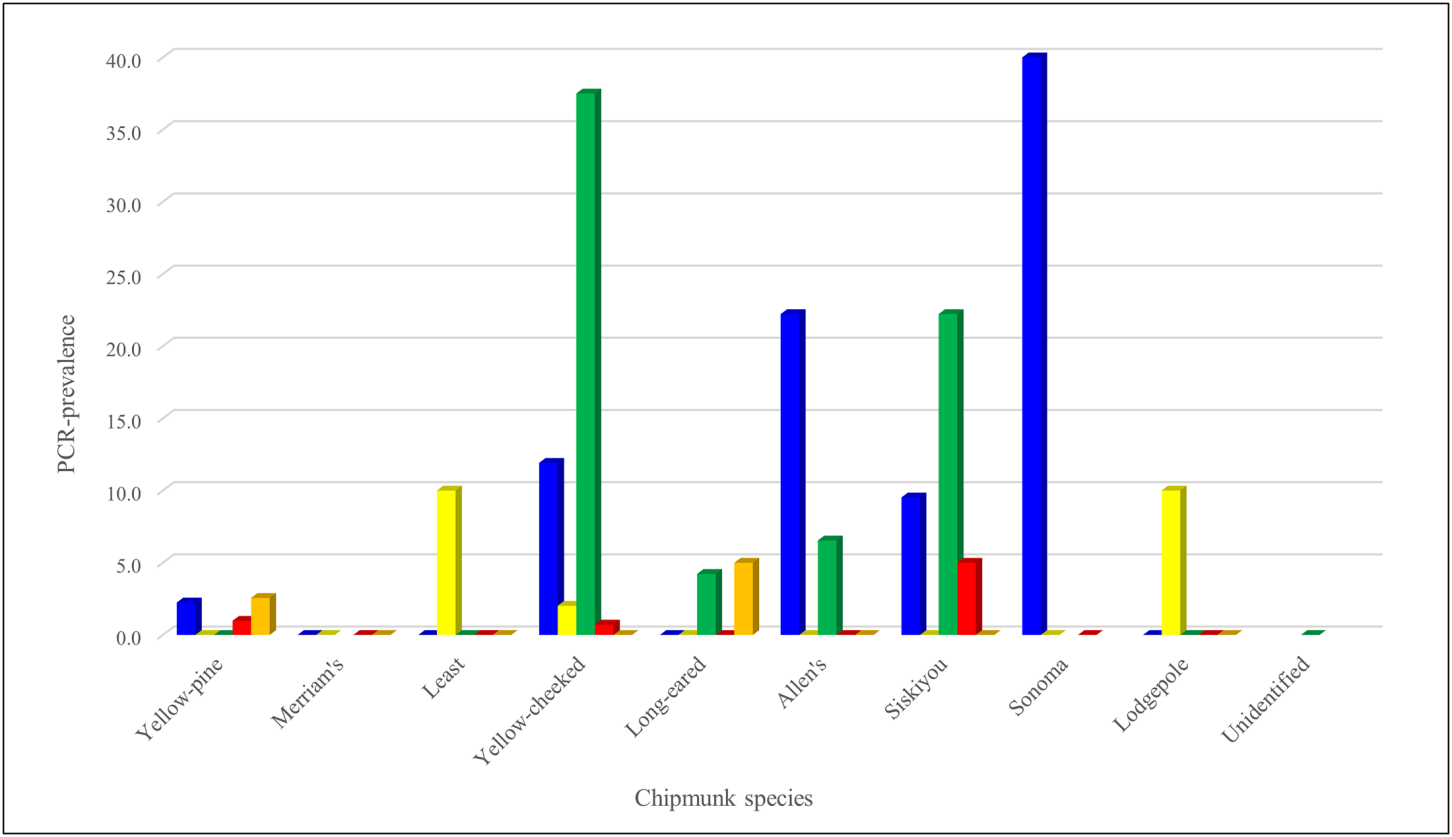
PCR-prevalence of vector-borne zoonotic pathogens in chipmunks (*Tamias* spp.) sampled throughout California between 2005 and 2015. *Anaplasma phagocytophilum* represented by blue. Relapsing fever *Borrelia* spp. represented by yellow. *Borrelia burgdorferi* represented by green. Spotted fever group *Rickettsia* represented by red. *Yersinia pestis* represented by orange.

**Fig 6:**
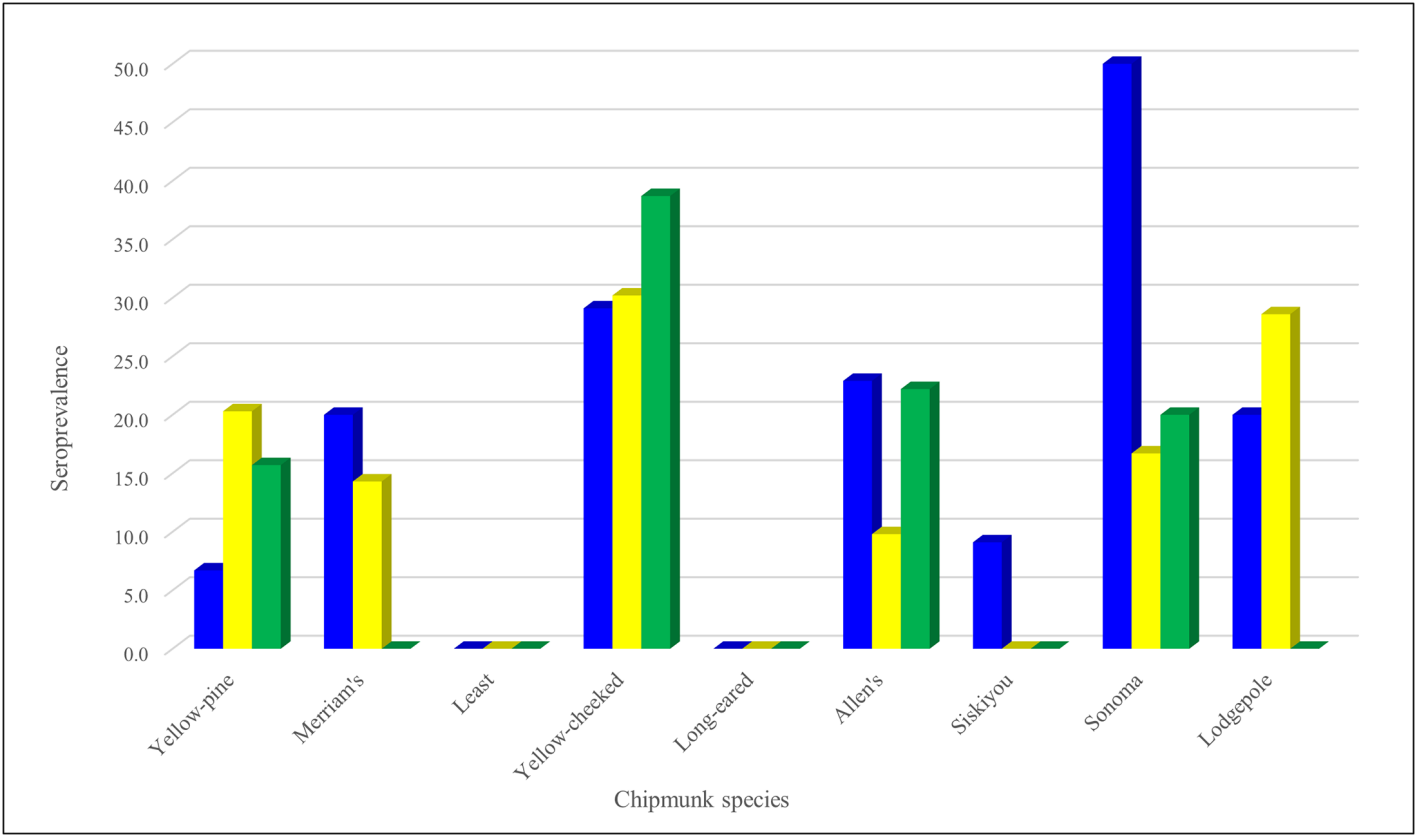
Seroprevalence of vector-borne zoonotic pathogens in nine species of chipmunks (*Tamias* spp.) sampled throughout California between 2005 and 2015. *Anaplasma phagocytophilum* represented by blue. *Borrelia* spp. represented by yellow. Spotted fever group *Rickettsia* represented by green.

All chipmunk species except for Merriam’s chipmunk were PCR-positive for at least one of the pathogens. Mirroring the regional analyses, active infection by *A*. *phagocytophilum* was highest in the Sonoma chipmunk (40.0%, 2/5) and differences in PCR-prevalence among species were significant (p = 0.037, Table 4, Fig 5). All species except the least and long-eared chipmunks had *A*. *phagocytophilum* antibodies, with the highest seroprevalence (50%, 3/6) in the Sonoma chipmunk; the differences found between species were significant (p < 0.001, Table 5, Fig 6). Active RF *Borrelia* spp. infection was found in the least, redwood (*T*. *ochrogenys*) and lodgepole chipmunks; differences among species were not significant (p = 0.065). However, highly significantly (p < 0.0001) more *B*. *burgdorferi* DNA was found in the redwood, long-eared, Siskiyou (*T*. *siskiyou*) and Allen’s chipmunks than in other species, with the redwood chipmunk having the highest PCR-prevalence (37.5%, 27/72). Although only the yellow-pine, lodgepole, redwood, Allen’s and Merriam’s chipmunks were *Borrelia* seropositive, differences in seroprevalence were not significant (p = 0.162). Three species were positive by PCR for SFG *Rickettsia* spp.: the yellow-pine, redwood and Siskiyou chipmunks; although differences in PCR among species were not significant (p = 0.589), differences in serology were (p = 0.015), with antibodies found in the yellow-pine, redwood, Sonoma and Allen’s chipmunks. Only two individuals were PCR positive for *Y*. *pestis*: one yellow-pine and one long-eared chipmunk.

**Table 4:** PCR-prevalence of vector-borne pathogens found in chipmunks (*Tamias* spp.) sampled throughout California between 2005 and 2015, stratified by chipmunk species.

	Anaplasma phagocytophilum*	Relapsing Fever *Borrelia*	Borrelia burgdorferi*	Spotted Fever Group *Rickettsia*	*Yersinia pestis*
	number positive/number tested (%; 95% CI)	number positive/number tested (%; 95% CI)	number positive/number tested (%; 95% CI)	number positive/number tested (%; 95% CI)	number positive/number tested (%; 95% CI)
**Yellow-pine**	2/89 (2.2; 0.6–7.8)	0/91 (0.0; 0.0–4.1)	0/61 (0.0; 0.0–5.9)	1/102 (1.0; 0.9–4.6)	1/39 (2.6; 2.3–11.6)
**Merriam’s**	0/9 (0.0; 0.0–29.9)	0/9 (0.0; 0.0–29.9)	n/a	0/10 (0.0; 0.0–27.8)	0/4 (0.0; 0.0–49.0)
**Least**	0/10 (0.0; 0.0–27.7)	1/10 (10.0; 6.6–35.6)	0/4 (0.0; 0.0–49.0)	0/4 (0.0; 0.0–49.0)	0/4 (0.0; 0.0–49.0)
**Yellow-cheeked**	36/302 (11.9; 8.7–16.1)	6/297 (2.0; 2.0–3.3)	27/72 (37.5; 31.2–45.1)	2/278 (0.7; 0.7–2.1)	0/69 (0.0; 0.0–5.3)
**Long-eared**	0/23 (0.0; 0.0–14.3)	0/22 (0.0; 0.0–14.9)	3/71 (4.2; 3.9–9.3)	0/23 (0.0; 0.0–14.3)	1/20 (5.0; 4.0–20.5)
**Allen’s**	9/129 (7.0; 3.7–12.7)	0/127 (0.0; 0.0–2.9)	7/107 (6.5; 6.0–10.1)	0/127 (0.0; 0.0–2.9)	0/77 (0.0; 0.0–4.8)
**Siskiyou**	2/21 (9.5; 2.7–28.9)	0/20 (0.0; 0.0–16.1)	4/18 (22.2; 15.6–38.6)	1/20 (5.0; 4.0–20.5)	0/13 (0.0; 0.0–22.8)
**Sonoma**	2/5 (40.0; 11.8–76.9)	0/5 (0.0; 0.0–43.4)	n/a	0/5 (0.0; 0.0–43.4)	n/a
**Lodgepole**	0/8 (0.0; 0.0–32.4)	1/10 (10.0; 6.6–35.6)	0/10 (0.0; 0.0–27.8)	0/12 (0.0; 0.0–24.3)	0/4 (0.0; 0.0–49.0)
**Unidentified species**	n/a	n/a	0/12 (0.0; 0.0–24.2)	n/a	n/a

n/a indicates no samples were available from that species for testing. CI = confidence interval

*A significant difference was found between species.

**Table 5:** Serological results of vector-borne pathogen surveillance of California chipmunks (*Tamias* spp.) sampled between 2005 and 2015.

	Anaplasma phagocytophilum*	*Borrelia* spp.	Spotted Fever Group *Rickettsia*
	number positive/number tested (%; 95% CI)	number positive/number tested (%; 95% CI)	number positive/number tested (%; 95% CI)
**Yellow-pine**	6/89 (6.7; 3.1–13.9)	14/69 (20.3; 17.0–26.7)	14/89 (15.7; 13.6–20.7)
**Merriam’s**	2/10 (20.0; 5.7–51.0)	1/7 (14.3; 8.1–45.8)	0/5 (0.0; 0.0–43.4)
**Least**	0/8 (0.0; 0.0–32.4)	0/8 (0.0; 0.0–32.4)	0/1 (0.0; 0.0–79.3)
**Yellow-cheeked**	77/265 (29.1; 23.9–34.8)	42/139 (30.2; 26.4–35.0)	72/186 (38.7; 34.6–43.3)
**Long-eared**	0/9 (0.0; 0.0–29.9)	0/8 (0.0; 0.0–32.4)	0/6 (0.0; 0.0–39.0)
**Allen’s**	27/118 (22.9; 16.2–31.2)	6/61 (9.8; 4.6–19.8)	24/108 (22.2; 15.4–30.9)
**Siskiyou**	1/11 (9.1; 1.6–37.7)	0/3 (0.0; 0.0–56.2)	0/8 (0.0; 0.0–32.4)
**Sonoma**	3/6 (50.0; 18.7–81.2)	1/6 (16.7; 3.0–56.4)	1/5 (20.0; 3.6–62.4)
**Lodgepole**	2/10 (20.0; 5.7–51.0)	2/7 (28.6; 15.0–57.3)	0/8 (0.0; 0.0–32.4)

CI = confidence interval

*A significant difference was found between species.

### Allen’s chipmunk

We examined one species, Allen’s chipmunk, which is distributed across the north-coastal super-region east into the Sierra Nevada. North-coastal individuals were significantly more likely to be seropositive for SFG *Rickettsia* spp. (p < 0.001) compared to individuals from the Sierra Nevada, while there were no significant differences between the seroprevalence of *A*. *phagocytophilum* or *Borrelia* spp., nor for any of the pathogens evaluated by PCR.

### Use of space

Five species of chipmunks were sampled from both the northern and western Sierra Nevada—yellow-pine, Allen’s, long-eared, least and lodgepole (Table 6). These five species were classified according to their reported use of vertical space: Allen’s and long-eared chipmunks were classified as strictly terrestrial; least and lodgepole chipmunks as arboreal; and the yellow-pine chipmunk classified as mixed, as it is typically terrestrial but also spends time in brush and small trees[37–39]. There was a significantly higher PCR-prevalence of RF *Borrelia* spp. (p = 0.013) in arboreal species, higher seroprevalence of *Borrelia* spp. (p = 0.037) in arboreal and mixed chipmunk species, and higher seroprevalence of SFG *Rickettsia* spp. (p = 0.003) in mixed chipmunks.

**Table 6:** PCR- and seroprevalence of vector-borne pathogens in chipmunks (*Tamias* spp.) sampled from the northern and western Sierra Nevada in California between 2005 and 2015 stratified by species’ reported use of space within their ranges.

Use of Space Designation	Mixed	Terrestrial	Arboreal
	number positive/number tested (%; 95% CI)	number positive/number tested (%; 95% CI)	number positive/number tested (%; 95% CI)
***Anaplasma phagocytophilum* PCR**	2/66 (3.0; 0.8–10.4)	5/61 (8.2; 3.6–17.8)	0/15 (0.0; 0.0–20.4)
***Anaplasma phagocytophilum* IFA**	5/68 (7.4; 3.2–16.1)	4/41 (9.8; 3.9–22.5)	2/15 (13.3; 3.7–37.9)
Relapsing Fever *Borrelia* PCR*	0/68 (0.0; 0.0–5.3)	0/60 (0.0; 0.0–6.0)	2/17 (11.8; 3.3–34.3)
*Borrelia* spp. IFA*	14/68 (20.6; 12.7–31.6)	0/23 (0.0; 0.0–14.3)	2/13 (15.4; 4.3–42.2)
***Borrelia burgdorferi* PCR**	0/40 (0.0; 0.0–8.8)	3/95 (3.2; 1.1–8.9)	0/12 (0.0; 0.0–24.3)
**Spotted Fever Group *Rickettsia* PCR**	1/79 (1.3; 0.2–6.8)	0/61 (0.0; 0.0–5.9)	0/13 (0.0; 0.0–22.8)
Spotted Fever Group *Rickettsia* IFA*	14/66 (21.2; 13.1–32.5)	0/38 (0.0; 0.0–9.2)	0/6 (0.0; 0.0–39.0)
***Yersinia pestis* PCR**	1/34 (2.9; 0.5–14.9)	1/37 (2.7; 0.5–13.8)	0/7 (0.0; 0.0–35.4)

Mixed includes Yellow-cheeked chipmunk, Terrestrial includes Allen’s and Long-eared chipmunks, Arboreal includes Least and Lodgepole chipmunks. IFA = Indirect immunofluorescent assay

*A significant difference was found between use of space categories

## Discussion

At least five vector-borne, zoonotic pathogens are present from the northern coast of California to the eastern Sierra in at least eight chipmunk species. Testing revealed substantial PCR- and seropositivity for both *Borrelia* spp. and *A*. *phagocytophilum*. Exposure to SFG *Rickettsia* spp. was also common, despite DNA in blood being extremely rare, presumably because the DNA is sequestered in the target cells for SFG rickettsiae, which are endothelium. Chipmunks from the northern coastal region were more likely to have evidence of vector-borne pathogen infection than were chipmunks from the Sierra Nevada. Our results are in agreement with previous, more geographically restricted, studies of vector-borne zoonotic diseases in California chipmunks [4, 5, 28, 40, 41] and highlight the importance of performing a study that spans numerous host species and geographical regions.

Here we used PCR and serology for most pathogens in order to evaluate both active infection of individuals as well as exposure and subsequent antibody production, which is a tool that gives useful insight into population responses to pathogens and herd immunity. The exceptions were *Borrelia* spp. PCR because ear tissue was not routinely collected in earliest sampling and *Y*. *pestis* serology because the antigen was unavailable. The poor correspondence between PCR and serology could be an outcome of reservoir hosts mounting meager antibody responses during infection with pathogens to which they are essentially tolerant, as described previously for tick-borne pathogens in woodrats [42]. *Borrelia burgdorferi* has clearly been shown to induce differential antibody responses across different reservoir species [43–45]. These results show that use of multiple tools is important to assess population status of hosts and infection.

As expected, there was abundant evidence of exposure to agents of Lyme disease and anaplasmosis in chipmunks from areas where humans are also found infected [46]. Redwood chipmunks are well-characterized reservoirs of *A*. *phagocytophilum*, with high PCR- and seroprevalence, relatively persistent infection for at least 4 months, and competence to infect the Pacific black-legged tick [47]. Numerous chipmunk species, and most abundantly the Allen’s chipmunk, have been found infected with *B*. *burgdorferi* in California, including *B*. *burgdorferi* sensu stricto and *B*. *bissettiae* in Siskiyou chipmunks, [23] as well as in chipmunks in Colorado [48]. In contrast to California where sciurids are reservoirs of both *A*. *phagocytophilum* and *B*. *burgdorferi*, chipmunks play a lesser role in the ecologies of these pathogens east of the Rocky Mountains, where white-footed mice (*P*. *leucopus*) account for the majority of infected ticks [6, 49]. The Siberian chipmunk (*T*. *sibericus*), which is becoming established in Europe, has been shown with higher tick infestation loads and *B*. *burgdorferi* sensu lato PCR-prevalence (primarily *B*. *afzelii*) than the recognized reservoirs [50].

The geographic distribution of cases of relapsing fever in California is much more restricted than for Lyme disease cases, reported most often in the Lake Tahoe and Mammoth Lakes regions of the Sierra Nevada [51]. Cases in California are typically associated with *B*. *hermsii*, transmitted by the soft tick *Ornithodoros hermsi*, which occurs at higher altitudes (450 to 2400 m) and feeds preferentially on squirrels and chipmunks [52]. These ticks can feed multiple times per stage and live more than a decade, ensuring long-term persistence of the pathogen in a site once contaminated [51]. Experimental challenge of a diversity of rodents by tick bite or injection resulted in spirochetemia in pine squirrels (*Tamiasciurus hudsonicus*), yellow-pine chipmunks, and meadow voles (*Microtus pennsylvanicus*) and clinical disease in the pine squirrels [53]. Statewide serosurveys have documented exposure in Allen’s chipmunk of 20% to a high of 47% in yellow pine and 57% in long-eared chipmunks in the Sierra Nevada [52], and PCR-based evidence of infection in a study focused near Big Bear Lake, CA was found in unidentified chipmunk species [5]. Although we detected a low prevalence of RF *Borrelia* in chipmunks, active infection was detected on the coast in redwood chipmunks, in addition to infection in least and lodgepole chipmunks in the Sierra Nevada. More work to differentiate among chipmunk species in their reservoir capacity is needed. This includes additional testing within fine spatial scale enzootic foci, reporting chipmunks to species, and additional experimental trials of multiple species of chipmunks.

We also found considerable evidence for *Rickettsia* spp. in chipmunks although most reactive chipmunks had antibodies but not DNA, making it impossible to determine which bacterial species they were exposed to. Rickettsiae are poorly sampled using PCR of blood because the target tissue is less accessible than blood, suggesting greater yield could be expected if sampling was done of whole carcasses. There are numerous co-circulating spotted-fever group rickettsial species, including *R*. *rhipicephali*, *R*. *montanensis*, and *R*. *rickettsii* [54–57]. Rodents can develop clinical disease with *R*. *rhipicephali* [58]. Chipmunks are reportedly susceptible to infection with *R*. *rickettsii* and an isolate was acquired from a yellow-pine chipmunk in Montana, although infection was not prolonged beyond a week [7, 59]. In theory, the low virulence rickettsiae could “immunize” small mammals against pathogenic *R*. *rickettsii* [60], while presence of one *Rickettsia* spp. in a tick can inhibit transovarial transmission of a second species [61].

Chipmunks are also known hosts of plague in California. Ground squirrels function as highly susceptible “amplifying hosts” and are responsible for most observed outbreaks, while voles (*M*. *californicus*), deer mice (*P*. *maniculatus*), and chipmunks experience less lethal disease but not prolonged infection and thus do not represent classical “reservoirs” [62]. Although the true mechanism by which plague persists in California is poorly understood, surveillance reveals natural hotspots in parts of the state including the Sierra Nevada where there is evidence of exposure in both rodents and carnivores in the Lake Tahoe area [28, 63–65]. An epizootic in chipmunks occurred shortly prior to a reported human pneumonic case contracted from a domestic cat [66]. Zielinski [65] also found that plague-positive pine martens (*Martes americana*) fed primarily on chipmunks, golden-mantled ground squirrels (*Callospermophilus lateralis*), and California ground squirrels. As we did not have access to antigen for serological surveillance, the very low PCR-prevalence is not unexpected given the limited duration of infection and relatively high rates of fatality in chipmunks. This suggests that seroprevalence would be low as well [62].

Are chipmunks particularly competent reservoirs or sentinels for vector-borne diseases and do the different species of chipmunks have distinct qualities that affect their reservoir competence? In coastal California, there is limited geographical overlap in species of chipmunks, with Sonoma chipmunks typically found somewhat inland, Merriam’s chipmunk primarily in chaparral, and redwood, Allen’s, and Siskiyou chipmunks distributed from south to north within distinct ranges bounded by large rivers [15]. Without experimental infections, it seems impossible to disentangle whether species are inherently more or less prone to infection from whether they are found in habitats that support vector-borne disease and are therefore more at risk of exposure to vectors and pathogens, such as the redwood chipmunk within moist coastal forests where ticks thrive [67, 68].

In contrast, Sierra Nevada chipmunks overlap, although fine-scale space use varies: Allen’s and long-eared chipmunks are almost exclusively terrestrial, yellow-pine chipmunks are primarily terrestrial but also commonly climb into brush and small trees up to 2m tall, while the least and lodgepole chipmunks exhibit both terrestrial and arboreal tendencies [37–39]. In theory, the presence of multiple species with slightly different space use could promote pathogen persistence if this diversity facilitates multiple routes of exposure to vectors and heterogeneity in development of herd immunity. Our findings of significantly higher levels of exposure to both *Borrelia* spp. and SFG *Rickettsia* spp. and higher PCR-prevalence of RF *Borrelia* spp. in chipmunk species that utilize both terrestrial and brush or arboreal habitats support this idea. One would also expect a pattern of increased pathogen diversity where there is increased chipmunk diversity, and our data did not support this, although the lack of such a relationship could represent a Type II statistical error. Interestingly, a previous comparison among species exposure to ticks revealed five different tick species (the highest richness found on any chipmunk species) on redwood chipmunks, one of the chipmunk species that occurs along the coast in areas typically not inhabited by any other chipmunk species [69]. We also showed that Allen’s chipmunks from the Sierra Nevada, where their range overlaps with several other chipmunk species, were significantly less likely to be exposed to SFG *Rickettsia* spp. than coastal Allen’s chipmunks where there is minimal range overlap among chipmunk species, also arguing against increased host diversity supporting more disease and suggesting that a species’ ecological importance may change depending on location.

Chipmunks are moderate sized rodents with longer lifespans than some reservoirs, such as white-footed mice, and are heavily infested with ticks and fleas in diverse parts of the world. They may achieve a higher population density than western gray squirrels, the western reservoir of *A*. *phagocytophilum* and *B*. *burgdorferi* [70, 71] but less than mice. As chipmunks often burrow underground, utilize old stumps and snags, forage on the ground, and seek refuge in canopy, sometimes even switching from a ground nest to a tree-cavity nest to raise pups [39], they may encounter ticks and fleas differentially at each of these different nodes. They may also share infected fleas and ticks differentially according to where other community members, such as tree and ground squirrels, are most likely to be present. As an example, *I*. *pacificus* ticks have been documented on tree trunks up to about a meter above ground [72] but have not been documented in canopies.

This study comprises the most far-reaching survey of vector-borne zoonotic diseases in California chipmunk species to date. The key findings provide several jumping-off points for future studies that will continue to increase our understanding of the complex ecological role that these small mammals play in disease maintenance and transmission in California.

## Supporting information

S1 Table Conventional PCR primers and TaqMan PCR primers and probes used to determine species of chipmunk or detect pathogen DNA in samples collected from California chipmunks (*Tamias* spp.) between 2005 and 2015. (DOCX)

S2 Table Original data. (XLSX)
