# Supplementary material for: Distribution and prevalence of vector-borne diseases in California chipmunks (Tamias spp.)
Source: PLoS One. 2017 Dec 12;12(12):e0189352. doi: 10.1371/journal.pone.0189352 (PMC5726628; doi:10.1371/journal.pone.0189352)
Supplement: S1 Table — (DOCX) [file pone.0189352.s001.docx]

S1 Table. Conventional PCR primers and TaqMan PCR primers and probes used to determine species of chipmunk or detect pathogen DNA in samples collected from California chipmunks (*Tamias* spp.) between 2005 and 2015.

| Target gene | Primers  (5’ to 3’) | TaqMan Probe  (5’ to 3’) |
| --- | --- | --- |
| Mammalian  *cytochrome b* | MVZ05: CGAAGCTTGATATGAAAAACCATCGTTG  MVZ16: AAATAGGAA(A/G)TATCA(C/T)TCTGGTTT(A/G)AT | n/a |
| *Anaplasma phagocytophilum* *msp2* | 903f: AGTTTGACTGGAACACACCTGATC  1024r:  CTCGTAACCAATCTCAAGCTCAAC | 939p-TTAAGGACAACATGCTTGTAGCTATGGAAG-GCA |
| *Borrelia burgdorferi*  16S rDNA | F: GCTGTAAACGATGCACACTTGGT  R: GGCGGCACACTTAACACGTTAG | 6FAM-TTCGGTACTA ACTTTTAGTTAA |
| Relapsing Fever *Borrelia* spp.  16S rDNA | F: GCTGTAAACGATGCACACTTGGT  R: GGCGGCACACTTAACACGTTAG | VIC-CGGTACTAACCTTTCGAT TA |
| Spotted fever group *Rickettsia* spp.  *gltA* | CS-F: TCGCAAATGTTCACGGTACTTT  CS-R: TCGTGCATTTCTTTCCATTGTG | CS-P: 6-FAM-TGCAATAGC AAGAACCGTAGGCTGGATG-BHQ-1 |
| *Yersinia pestis pla* | 109F: GGAGGTACTC-  AGACCATTGATAAGAAT  209R: AGACC CGCCGTCAC  AGTATAA | 159p-  CGGAGATGCTGCCGGTATTTC CAATAAAA |
